# Ewing's tumour, a report on 27 cases

**Published:** 2008-05

**Authors:** Vyankat G Vohar

**Affiliations:** Department of Radiology, Tata Memorial Hospital, Mumbai, India Previously Published, August 1949, Vol 3 No 3

Ewing in 1921 first described a distinct malignant tumour occurring in the soft tissues of the skeleton and called it “endothelial myeloma”. It is described in the literature as a relatively rare disease. Our records show a total of 27 cases of Ewing's tumour between the years 1941 to 1947. Of the total number of 9985 patients examined at the Tata Memorial Hospital between the period 1941 to April, 1945, 9640 were found to suffer from malignant disease of various tissues of the body. Sixty-five of these were primary malignant bone tumours: sixteen of these primary bone malignancies were Ewing's sarcoma. Thus of the total number of 27 Ewing's tumours seen to date, 16 were upto 1945 and 11 were seen between 1945 to the end of 1947. Tables 1, 1a and b shows the incidence of age, sex, duration of symptoms and site of origin of the 27 cases collected from our files. A positive microscopic diagnosis of Ewing's tumour was obtained in 25 cases after aspiration or formal biopsy and in 2 cases after autopsy; in both autopsied cases the pathologist reported the adrenals as normal.

**Table 1:** 

Case No.	Age	Sex	Duration on addmission	Bone involved	Trauma
598	20	Male	9 months	Left femur (amputated)	Lower end
2316	23	Female	1 year	Left pubic bone	
2498	44	Male	9 months	Right ribs	
3561	50	Male	6 months	Right femur - mid-shaft	
4066	30	Male	5 months	Left femur - (neck)	
4155	6	Female	8 months	Right tibia and lower end femur	
5004	30	Male	4 months	Left femur upper	
5452	12	Female	4 months	Right femur	
5616	34	Male	4 months	Right upper humerus - epiphysis involved	
5814	16	Male	3 years removed recurrence 3 months	Ribs	
7124	13	Male	4 months	Right acetabulum	
7361	24	Male	2 years	Right pubic bone	
7470	10	Male	4 months	Right mastoid	
8356	4	Male	5 weeks	Frontals	
8665	15	Male	7 months	Left ilium	
9017	6	Female	2 months	Right femur	
9615	30	Male	1 year	Left ilium	
10519	10	Female	6 weeks	Right fibula	
12302	11	Female	3 months	Left upper tibia - (metaphysis)	
13134	28	Male	9 months	Right ilium	
13161	20	Male	10 months	Right humerus - epiphysis: glenoid and joint	
13250	23	Male	3 months	Right ilium	
G 496	16	Male	8 months	Left fibula	Lower third
G 2293	26	Female	6 months	Right 3^rd^ rib	
G 2830	25	Male	1 year	Left upper end fibula	
G 3156	8	Female	2½ months	Left femur	
G 3354	16	Male	4 months	Frontal bone

**Table 1a:** 

Age in years	No. of cases	Years
1–10	6	(4, 6, 6, 8, 10, 10)
11–20	9	(11, 12, 13, 13, 15, 16, 16, 20)
21–30	9	(23, 23, 24, 25, 26, 28, 30, 30)
31–40	1	(35)
41–50	2	(41, 50)

**Table 1b:** Duration of symptoms

Period in months	No. of cases
1–3	6
3–6	9
7–9	6
10–12	4
Above 12	2

The disease is more common in men than in women. Twenty of 27 patients were males and 7 females. Although no age seems to be exempt, the disease usually affects persons in the second and third decades of life. Nineteen of our patients were within this age period. The youngest was years of age, the oldest being 50. The duration of disease in all our cases, except two, was within 1 year. In one patient, the duration of symptoms was three and a half years.

## Site of Origin

**Figure 1 (A, B):**
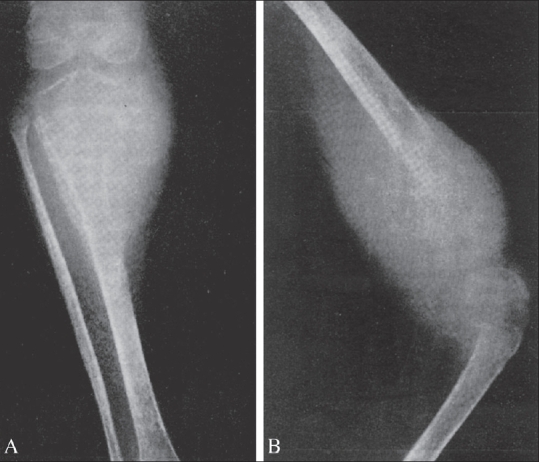
Ewing's sarcoma. The same patient shows involvement of the proximal tibia (A) and distal femur (B)

The bones of the pelvic girdle and the lower extremities are the most common sites of disease. It may, however, affect any part of the skeleton, including the vertebrae, ribs, skull and even the small bones of the hands and feet. In 19 of our cases the disease was located in the pelvic girdle and the lower extremities. None of our series had involvement of the vertebrae or the small bones of the extremities. When occurring in long bones, the disease was believed to affect only the shafts. In our material, the ends of long bones were often involved. Thus we had three cases with involvement of the ends of the femora, two in the upper end of the humerus, two in the upper end of the tibia and one in lower end of the fibula. In one patient, the lower end of the femur with the corresponding upper end of the tibia was involved [Figure 1]. The disease is believed to start in the Haversian canals, subperiostially, or in the medulla. The extent of medullary involvement is usually greater than shown in the skiagram. This is so because the disease is seen in the radiogram only when it affects the medullary side of the cortex and produces osteolysis or sclerosis. As the disease advances, it destroys the cortex, spreads underneath the periosteum, destroys it and produces variable amount of invasion of soft tissue. Thus a large soft tissue swelling - as distinct from an osseous swelling - is a sign of advanced disease with cortical destruction. Potozky and Fried have reported a group of cases of Ewing's tumour with a large soft tissue mass as a presenting sign in which the radiogram gave no indication of the osseous origin. Autopsy examination however showed involvement of the surface layers of the bone. In this connection it is worth mentioning, that the soft tissues of the extremities are stated to be often the site of a round cell tumour, histologically resembling Ewing's tumor and showing a similar radio-sensitivity.

## Clinical Data

Clinically, it usually presents with the well-known inflammatory complex of fever, pain, swelling and leucocytosis. A history of fever is not present in all. Pain is a constant feature. It varies from moderate to very severe, is often intermittent in character, and is more marked at night. Pain in orther parts of the skeleton is the earliest indication of metastasis. With this early metastasis the radiogram is usually negative. Six of our patients gave a definite history of trauma. The rest had either no trauma or no definite awareness of it. Trauma seems to have no direct causal relation to bone tumours, for were it so the incidence of bone tumours should be much higher than it actually is, and yet it is difficult to escape the impression that trauma in some cases is an exciting cause. Enlargement of the nearest draining lymph nodes is often present. Five of our patients had enlarged nodes, three in the groin, one in the neck and one in the popliteal fossa. Two of our patients (Nos. 1230 & 6545) had rounded masses in the upper abdomen. In one of these, the bone lesion was in the upper tibia, and the other in the mid-femoral region. The abdominal masses were noticed two and five months respectively after the bone lesion was seen. In both these cases, the possibility of a primary (clinical) neuroblastoma cannot be excluded. The disease progresses fairly rapidly metastasizing rapidly to the nearest lymph nodes, the lungs and the distant bones and leading to a fatal termination with-in a few months to a couple of years.

## Laboratory Findings

All of our patients, except one, showed leucocytosis, the white blood cell (WBC) count varying between 8 to 20 thousands. One patient had a WBC count of 4000. Seven patients had moderate to severe anaemia, the red blood cell (RBC) count being below 4 millions on admission. In the rest, the count was about 4.5 millions. The serum calcium varied round 10 mg%; the phosphorus values were within normal limits. Alkaline phosphatase varied between 1.5 to 5.5 B Units. Acid phosphatase values were normal. Kahn's test was negative in 18 patients; in the rest, the test was not done.

In osteogenic sarcoma the values for serum alkaline phosphatase were between 10 to 16 B Units. The higher values of alkaline phosphatase in osteogenic sarcoma could be utilised as an additional diagnostic help for differentiation from Ewing's sarcoma.

## Roentgen Manifestations

**Figure 2 (A, B):**
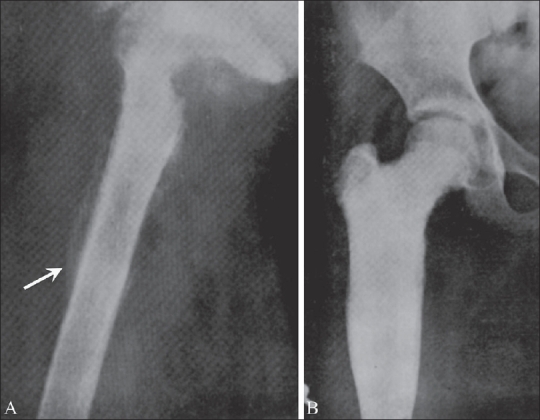
Ewing's sarcoma of the proximal femur. Pre-irradiation (A) oblique radiography shows an “onion-peel” appearance (arrow) with destruction of the marrow cavity. Post-irradiation (B), there is marked sclerosis and cortical thickening

Roentgen examination of bone tumours is now established as an indispensable diagnostic procedure. For some time, fixed roentgen criteria were ascribed to different varieties of tumours. For instance the “sun-ray” appearance was considered characteristics of osteogenic sarcoma, the “onion peel” appearance for Ewing's tumour [Figure 2], “soap bubble” appearance was considered very typical of “giant cell tumours”. A definite diagnosis of the cellular nature of the tumour was given by the radiologist. Now the impression is gaining ground that these are so-called characteristic appearances are, after all not so “characteristic”. Roentgen examination can give a definite evidence of malignancy in advanced cases. In early cases, even this is not always possible. Malignant bone lesion is a progressive disease. Roentgen ray appearance will therefore vary with the activity of the neoplasm and the response of the stroma. As Macdonald and Budd say “the appearance of a bone tumour at a given time depends upon the balance between the neoplastic activity (osteolysis) and the degree of cortical and medullary reaction (sclerosis) as well as the absence and extent of ossification and calcification.” The characteristic appearance in a radiogram is thus a reflection of the activity and growth. These appearance would not always indicate the tumour type. To attempt to give a definite diagnosis of the cellular make up from the roentgengram alone is to fail to recognise the limitation of the method. This can only bring the roentgen diagnostic method to disrepute. Table 2 gives an analysis of the main roentgen findings in Ewing's tumour. We have followed the method of presentation of Swensen with slight modification.

**Table 2:** Analysis of roentgen appearances

Case No.	Cortical condensation	Osteolysis	“Onion peel” reaction	Other periosteal reaction
598		X-ray not available		
2316	0	+	0	0
2498	0	+	0	+
3561	0	+	0	+
4066	0	+	0	+
4155	0	+	0	+
5004	+	+	+	+
5452	+	+	+	+
5616	0	+	0	Slight
5814	0	+	0	0
7124	0	+	0	0
7361	0	+	0	0
7470	0	+	0	0
8356	0	+	0	+
8665	+	Scanty	+	0
9017	0	+	+	+
9615	0	+	0	0
10519	0	+	0	0
12302	+	+	+	0
13134	0	+	0	0
13161	+	+	0	Scanty
13250	0	+	0	0
G 496	0	+	+	0
G 2293	0	+	0	0
G 2830				
G 3156	0	+	0	+
G 3354	0	+	0	+

**Figure 3 (A, B):**
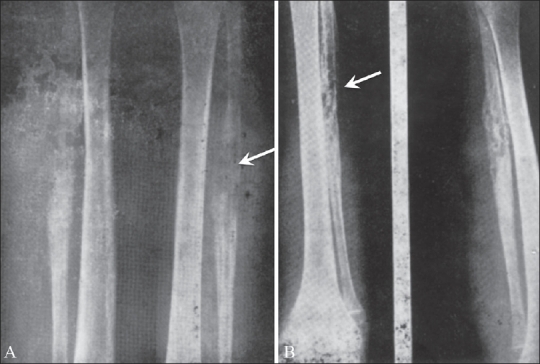
Ewing's sarcoma of the fibula. Pre-irradiation radiographs (A) show a destructive, osteolytic lesion of the fibular diaphysis (arrow). Post-irradiation (B) radiographs show a mottle appearance with evidence of healing (arrow)

**Figure 4 (A, B):**
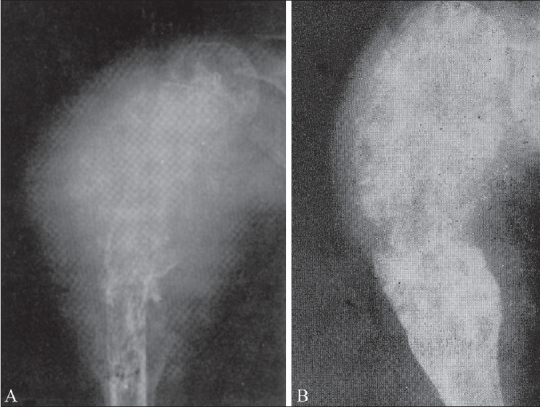
Ewing's sarcoma of the humeus. Pre-irradiation radiograph (A) shows a destructive lesion with a “honey-comb” appearance. The post-irradiation radiograph (B) shows a mottled appearance with sclerosis and residual areas of osteolysis

Osteolytic destruction of the involved bone is the most frequent radiological finding [Figure 3]. Twenty-one of 24 of our cases whose radiograms were available showed marked osteolysis; in the remaining 3 cases there was scanty osteolysis. Five of the patients showed variable associated cortical condensation. Osteolytic changes take on various forms. In flat bones, this may appear as a complete homogenous lysis or a stippled rarefaction. In long bones, the osteolytic changes are seen in various stages and location. This may manifest as subcortical erosion, or as destruction of the medullary side of the cortex and widening of the medulla. When affecting the Haversian canals various degrees of mottled rarefaction of the cortex is seen. In other cases, the affected shaft shows a “honey- comb” appearance [Figure 4]. Occasionally the whole of the involved bone is completely destroyed with thin strips of bone lying in the tumour mass, as faint remnants of the original cortex.

**Figure 5 (A, B):**
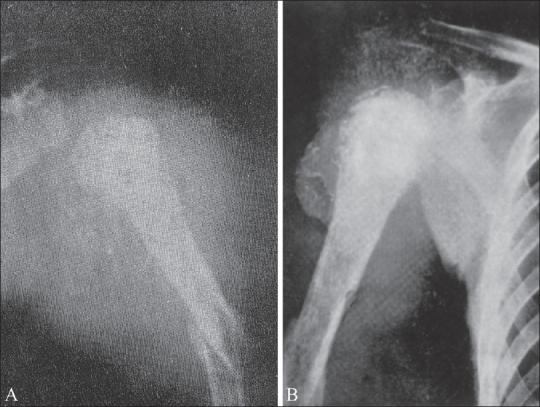
(A, B): Ewing's sarcoma of the humerus. Pre-irradiation radiograph (A), shows a lesion with marked condensation of bone with a large soft tissue swelling. Post-irradiation (B) radiograph shows regression of the soft tissue and bone condensation

Condensation of bone, when present may occur intracortically or subperiosteally [Figure 5]. Intracortically condensation is due to diffuse deposition of bone among the trabecular structure. This produces increased density of the cortex and sometimes narrowing of the medullary canal. Unlike osteogenic sarcoma, there is no bone formation by the tumour cells. Only reactive bone is produced.

**Figure 6 (A, B):**
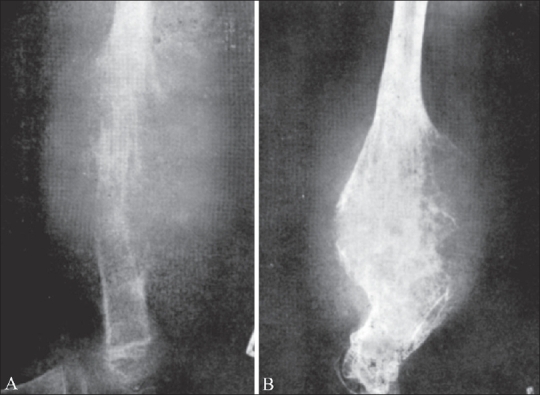
Ewing's sarcoma of the femur. Pre-irradiation radiograph (A) shows a large, destructive lesion, showing a bit of a “sun-ray” appearance. Post-irradiation radiography (B) shows bone condensation and healing

**Figure 7:**
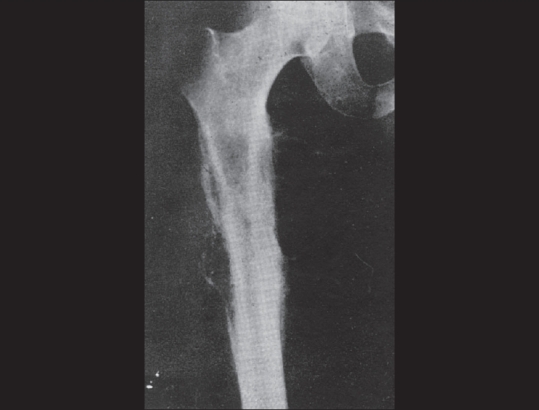
Ewing's sarcoma of the proximal femur. The plain radiograph shows a heterogeneous appearance with a bizarre pattern of periosteal and subperiosteal reaction

A variety of patterns is presented by sub-periosteal new bone formation. The well known “onion peel” appearance is only occasionally seen in Ewing's tumour. On the other hand, it is sometimes seen in chronic pyogenic or syphilitic osteomyelitis. Its presence therefore does not confirm nor its absence exclude the diagnosis of Ewing's tumour. Sometimes the subperiosteal new bone is laid down in thin irregular striations placed perpendicular to the shaft, resembling the “sun-ray” appearance [Figure 6], thought to be characteristic of osteogenic sarcoma. The “sun-ray” arrangement of subperiosteal new bone was seen in two of our patients in the frontal bones. The involved bone sometimes shows a combination of both the “onion peel” and “sun-ray” pattern of new bone formation. More often, there is an irregular bizarre pattern of subperiosteal reaction [Figure 7].

**Figure 8:**
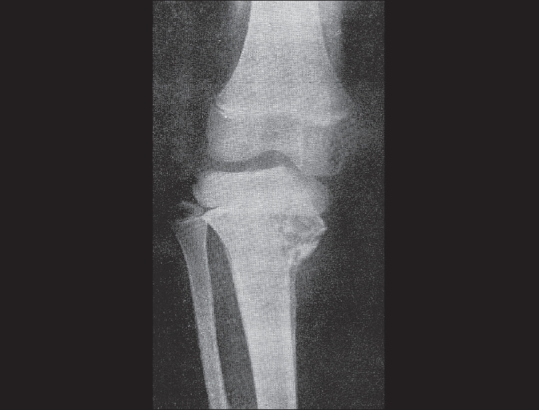
Ewing's sarcoma of the proximal tibia. The plain radiograph shows an irregular, osteolytic lesion in the medial aspect of the proximal tibial metaphysis. This was suspected to be osteomyelitis, but turned out to be a Ewing's sarcoma on biopsy

From the radiogram alone, a differentiation of Ewing's tumour has to be made from a variety of bone lesions. These include osteomyelitis [Figure 8], osteolytic form of osteogenic sarcoma, metastatic bone deposits, reticulum cell sarcoma etc.

## Histopathology

It is now widely accepted that a positive diagnosis of a malignant bone lesion requires the combined efforts of a surgeon, the radiologist and the pathologist. The pathologist is obviously the final arbiter. And yet often the pathologist does not arrive at a positive diagnosis from his microscopic picture alone without reference to the skiagram and the clinical picture. Histologic diagnosis is not written on the slide. The microscopic appearance has to be interpreted. Leaving aside the personal factor, then the microscopic appearance, particularly with Ewing's tumour admits of different interpretation. A fair amount of polemical literature has collected about the nature of this bone lesion. Although most experienced oncologists following Ewing, accept this lesion as a distinct disease process, an emphatic opposition to such a view is voiced by some pathologist of reputed e.g, Willis, Colville, and Steruburg. They regard all cases of Ewing as “precocious” metastatic bone lesions from an occult neuroblastoma either in the adrenal or the sympathetic ganglia. However Willis in his latest book “Pathology of Tumours” published in 1948, says “some cases at least of Ewing's syndrome are due to clinically obstructive metastasis of neuroblastoma in long bones”(p. 864). One could ill afford to hold a dogmatic opinion on this count. The only course open is that of taking the “majority” view as a working hypothesis, always keeping in mind to look for data which go to disprove such hypothesis.

The cytological appearance of Ewing's tumour is fairly constant. It shows “sheets” or groups of cells, with varying amount of fibrous strands in between. The cells are usually round with some variation in size. Cell walls are indistinct with scanty cytoplasm and large hyperchromatic nuclei. Nuclei have well-defined margins with scattered chromatic material. Sometimes the cells are polygonal when closely packed. Cells may be arranged along blood vessels and the RBCs may be separated from the tumour cells only by endothelium and delicate strands of reticulum, the so-called perithelial arrangement.

Occasionally the cells are arranged in a “pseudo-rosette” formation. These are structures with a circular arrangement of cells, the nuclei of the cells being situated at their outer poles. Sometimes argyrophil reticulum fibres are seen scattered between groups of cells.

These microscopic appearances form the basis of a diagnosis of “Ewing's Tumour”. This picture differs from the original description of the cell type by Ewing. He described it as “a small polyhedral cell with pale cytoplasm, a small hyperchromatic nucleus, and a well-defined cell wall.” More recent reports (Stout and Jaffe) however described cells to have a large nucleus, scanty cytoplasm and indistinct cell walls.

Jaffe and Leichtenstein do not ascribe any diagnostic significance to the “perithelial” arrangement of cells. They state that haemorrhage into the neoplastic tissue is accompanied by ingrowth of blood vessels. Many of these vessels becomes surrounded by tumour cells. According to them, the “vessel spaces are not lined by tumour cells.” Besides, they state that a similar perivascular arrangement of tumour cells is seen in other sarcomas when focal areas have undergone extensive haemorrhage.

The “pseudo-rosette” appearance in Ewing's tumour is cause of much controversy. Similar structures are seen during the development of the sympathetic nervous system and frequently in neuroblastomas arising from the adrenals and sympathetic system. Proper staining of these however will show neurites protruding from the central ends of the cells. The central cores will show fibrillar or granular appearances. Jaffe and Leichtenstein state that the cores of the so-called rosette arrangement in Ewing's tumour do not show a fibrillar or granular appearance, but are made up of degenerated nuclei and necrosis, the radiating arrangement of cells being explained on a purely physical basis. Stout, himself is not satisfied with this explanation. He says ‘orientation of the nuclei towards the peripheral pole has a teleological aspect which suggests an activity other than pure degeneration. Consequently, their presence in the Ewing's tumour remains for me unexplained and in need of further elucidation”

Colville and Willis (1933) and Willis (1940) because of close resemblance of the cytologic picture of Ewing's tumour with metastasis from sympathetic neuroblastoma, warned against making a diagnosis of Ewing's tumour from clinical and biopsy data. Only carefully executed autopsies can exclude neuroblastoma. Without this a diagnosis of Ewing's sarcoma is not justified.

A perusal of the available literature on the subject shows that most experienced oncologists (Stout, Jaffe etc.) while accepting the weighty objection of Willis et al are inclined to regard Ewing's sarcoma as a disease entirely separate from metastatic bone lesions from sympathetic neuroblastoma, despite absence of autopsy examination in most of their cases and on the basis of the biopsy microscopic picture alone. Other lesions which often require to be differentiated from Ewing's tumour are reticulum cell sarcoma, lymphocytic lymphoma, and Hodgkin's disease. Sometimes bone metastasis from a silent highly malignant carcinoma may be the source of error, when miscroscopically it presents a uniform pattern of round cells.

## Roentgen Therapy

Ewing's tumour, like reticulum cell and lipogenic sarcoma, is highly radio-sensitive. High radio-sensitivity in Ewing's tumour means a rapid initial regression of the tumour mass and not a complete control of the malignant process nor even in most cases an extended survival. With this high sensitivity of the tumour, theoretically it should be possible to obtain an extended survival if not complete cure by early adequate irradiation. Yet few “cures” are reported. The tumour is highly malignant and rapidly spreads to distant parts. The disease generally extends more along the medulla than is shown by the radiogram, so that treatment of the tumour area as shown by the radiogram would be inadequate. A large portion of the adjacent normal bone should be included in the field.

In most cases this would require inclusion in the field of the whole of the bone. A tumour doze of 4500r at least is necessary. Woodard and Coley have recently reported that a tissue dose of 3000 to 5000r could control the tumour for varying period; after this dosage a large number of viable tumour cells were still present. They report complete destruction of all tumour cells with tumour dose approaching 6000 to 7000r. Two of their patients so treated are living after eleven years. This shows that despite its apparent sensitivity, for complete control the tumour requires doses as high as does a squamous cell tumour. This would necessarily entitle much damage to the normal bone. It is not generally realised that adult normal bone is more vulnerable to irradiation than surface epithelium. Besides it is now definitely established that absorption of penetrating radiations by bone is higher than in soft tissues. Very high tumour doses necessary to control Ewing's tumour will permanently damage the adjacent normal bone which has to be included in the treatment field.

**Figure 9 (A, B):**
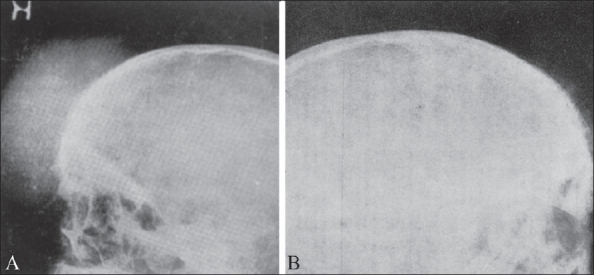
Ewing's sarcoma of the frontal bone. The pre-irradiation plain radiograph (A) of the skull shows condensation of bone in the frontal region with a large soft tissue swelling. The post-irradiation radiograph shows virtually complete regression of the soft tissue

Under irradiation the initial regression of Ewing's tumour is often amazingly rapid, with control of pain and marked reduction in soft tissue swelling [Figure 9]. There is noticeable improvement in the general condition and the mental attitude of the patients. New bone formation becomes visible on the radiogram from about the third week of the commencement of treatment. During the 4^th^ to the 6^th^ week dense new bone becomes definitely established. The irregular cortical margins are smoothened and layers of dense bone are deposited along the shaft which may show hardly any evidence of the malignant process.

All case of Ewing's tumour do not show this high sensitivity. Some are definitely resistant. Two of our patients gave very poor response to irradiation. One of these, an eleven year old girl with involvement of the upper tibial metaphysis had no reduction in pain or swelling. The patient died within two months with lung metastases. An opportunity was afforded to us for noticing this radioresistance while treating two almost identical cases referred to us from the British Military Hospital in Bombay. The right ilium was the seat of disease in both, with nearly similar roentgen appearance. Both were treated during the same period with identical deep therapy technique. One of these showed excellent response. Bone regeneration was seen within four weeks. The second patient had no reduction in pain. Soft tissue swelling was slightly reduced and the patient gradually went down during treatment.

Our results of radiation treatment so far as extended survival is concerned are extremely disappointment. Twenty-two patients came up for X-Ray treatment. All of these except one, were treated with 200 kV. 0.5 mm Cu plus 1 mm. AL filter, 50 cm F.S.D; and one was treated with 400 kV. 4 mm Cu plus 1 mm. AL filter 50 cms. distance. The doses ranged from 2400r (air) on each of the ports in cases of long bones, to 3000 and 3500r on each of two ports in flat bones. Three of the treated cases were seen alive without metastases for 1 year; after that there was no “follow up”. Three were seen up to 6 months without disease; then no follow-up. Six patients never came for follow-up after completion of treatment. One lived for two years but with metastases in various parts of the body. We chased the disease from place to place with X-ray treatment, each time with excellent symptomatic improvement, till finally, the patient succumbed to extensive metastases in the spine. Three patients, treated within the last four mouth of 1947 are still living - two without metastases and one with metastases.
